# Digitalisation in the WFP fresh food voucher programme: a pilot study from rural Amhara region, Ethiopia

**DOI:** 10.3389/fnut.2023.1217794

**Published:** 2023-11-06

**Authors:** Fazal Dad, Filippo Dibari, Aweke Kebede, Emma Lefu, Tafara Ndumiyana, Blessing Butaumocho

**Affiliations:** ^1^WFP Ethiopia CO, Regional Bureau Nairobi, Addis Ababa, Ethiopia; ^2^WFP Regional Bureau Bangkok, Bangkok, Thailand; ^3^WFP Bangladesh CO, Regional Bureau Bangkok, Dhaka, Bangladesh

**Keywords:** fresh food voucher, unstructured supplementary services data (USSD), food purchasing pattern and point of sale, stunting (POS), World Food Programme (WFP)

## Abstract

**Introduction:**

Malnutrition continues to pose a major challenge to human well-being around the world. In Ethiopia, 39% of children <5 years are stunted, with peaks in northern regions of the country such as Amhara (54.8%). Very few (2%) children in the region achieve the minimum dietary diversity and only a minority (27%) belong to households that can afford a nutritious diet. To tackle the high stunting rate, diets high in fruits and vegetables are widely recommended to improve dietary diversity. Programmes leveraging fresh food vouchers can be used to support vulnerable groups with malnutrition and limited affordability. Cash-based transfer (CBT) programmes have repeatedly been shown to improve child growth and increase household food security and dietary diversity. This study is part of the World Food Programme (WFP) intervention regarding a stunting reduction rural programme of restricted CBT for improving dietary diversity in households with children under 2 years of age and pregnant and lactating women.

**Methods:**

A community- based pilot study to assess the itemised foods purchased by beneficiaries was conducted in the four most accessible woredas of the Amhara region of Ethiopia. A total of 556 beneficiaries and 12 active retailers were selected randomly from 10 rural markets in the targeted woredas. A point of sale (POS) system was used to collect the itemised food prices and amounts of food procured by the beneficiaries.

**Results and Discussion:**

Approximately 51, 35, and 15% of the beneficiaries purchased vegetables, fruits, and eggs, respectively. Prices, taste preferences of children, and shelf life determine the purchase of certain food items. The average food expenditure was 49 Ethiopian Birr (ETB; US$ 0.94), representing the 63 and 37% daily and monthly requirements, respectively, for affordability. The higher increase in prices of some food items might be due to their seasonality. Almost half of the Fresh Food Voucher (FFV) beneficiaries were purchasing and consuming vegetables. The finding indicates that the WFP fresh food voucher programme contributes 63% (49 ETB, US$ 0.94) and 37% (837 ETB, US$ 16.1) of the daily and monthly needs of affordability, respectively, for a diversified nutritious diet. The use of Unstructured Supplementary Service Data (USSD) technology in the WFP digital voucher under the Fresh Food Voucher (FFV) project was effective at collecting itemised prices of foods purchased by the beneficiaries. The point of sale (POS)^1^ system can be scaled up under the Fresh Food Voucher (FFV) programme so that the digital voucher can contain the itemised food prices. Timely data from the point of sale could be used for timely Social Behaviour Change Communication (SBCC) development to improve dietary diversity.

## 1. Introduction

In Ethiopia, malnutrition contributes 39% of child stunting at costs greater than 16% of the national annual gross domestic product (1, 2). The burden of stunting is particularly high in both Amhara (46%) regions, and children in rural areas are more vulnerable than those in urban areas (3, 4). As few as 2% of children achieve the minimum dietary diversity (5), with 73% of households unable to afford a nutritious diet (6).

These findings are backed up by more recent evidence from the Fill the Nutrient Gap (FNG)^2^ Analysis of Ethiopia (6), which shows that dietary diversity and meal frequency are particularly low in children under 2 years of age and only one in four households can afford a nutritious and diversified diet. Since fruits and vegetables are mostly procured at markets, rather than being produced, in urban areas, the cost of a nutritious diet has likely increased (6) due to the disruption of the supply chain as a result of the COVID-19 pandemic. According to a study by the International Food Policy Research Institute (IFPRI) in Ethiopia, the affordability of a diversified and healthy diet exceeded household income by 53% (7). Poor food affordability shifts household consumption away from diverse foods and towards staple foods.

The Productive Safety Net Programme (PSNP) in Ethiopia provides a food basket that partially covers the nutritional requirements of recipients. Between 2018 and 2021, the World Food Programme’s Fresh Food Voucher (FFV) programme ‘topped up’ the food basket of 27,000 vulnerable households. The eligible households were those with pregnant and lactating women and children under 2 years of age.

The FFV programme was designed to promote growth and reduce stunting prevalence by improving dietary diversity and quality for pregnant women, lactating mothers, and children 6–23 months of age. Within the programme, a digital voucher is sent monthly via SMS message to the mobile phone of a member of each recipient household. The SMS message includes a code that can be used to procure fruits, vegetables, and eggs as a group from small retailers in rural markets. Recipient households send the SMS-based code to the mobile phone of a retailer, who is also enrolled in the programme. This initiates payment for the transaction and only requires a simple analogue mobile phone, without the need for an internet connection. Selected retailers were operating at a small scale with limited capital, technology, and no permanent infrastructure. They were not using any point of sale (POS) solutions to record itemised sales data.

The objective of this research is to show how digitalisation in the fresh food voucher programme facilitated the timely transfer of vouchers from the World Food Programme (WFP) to financial service providers and then to retailers for beneficiaries to redeem the itemised foods based on their entitlement. The WFP decided to collect data on the fresh food items procured by beneficiaries (what the beneficiaries are buying, in what quantity, and at which price) using Unstructured Supplementary Service Data (USSD) technology. Therefore, this qualitative study is aimed to evaluate the effectiveness of USSD technology in the given context of rural Ethiopia.

## 2. Materials and methods

### 2.1. Study location and sampling

A community-based pilot study was conducted from September to December 2020 to assess the itemised foods purchased by recipients in the four most accessible woredas (districts) of the Amhara region of Ethiopia. A total of 556 recipients and 12 active retailers were randomly selected from 10 rural markets in the targeted woredas. Itemised foods purchased by each of the 556 recipients were recorded for each month during the study period.

### 2.2. Study design and setup

The innovative Unstructured Supplementary Service Data (USSD) system was developed to allow Fresh Food Voucher (FFV) market retailers to transmit data about itemised sales, alongside data on the redemption of digital vouchers. The retailers pre-set food prices using the same technology and entered the quantity upon purchase. The system calculated the total cost based on the quantity and type of food purchased and sent an invoice to recipients via mobile SMS.

### 2.3. Unstructured supplementary service data technology

Unstructured Supplementary Service Data (USSD) is an interactive menu-based technological communication protocol available on all Global System for Mobile (GSM)-enabled mobile devices. The Unstructured Supplementary Service Data (USSD) technology works on any mobile phone model, including analogue phones, and does not incur additional costs to retailers or recipients. It is a wireless network that uses SMS messages with mobile phones and stores data. It enables a real-time connection between a phone and a software application. The technology reduces the time and resources needed to give money to beneficiaries to buy fresh food. Moreover, it helps to restrict the type of food that the household buys. If money is directly transferred to beneficiaries, they could buy whatever they want or even use the money for other household priorities. However, here, the type of food they buy is restricted to that which helps prevent wasting amongst the target group only.

### 2.4. World food programme fresh food voucher composition

The food voucher included locally available and locally produced foods, with consideration of the nutritional requirements for pregnant and lactating women (PLW) and children 6–23 months of age. The voucher provides a fixed amount of cash that recipients can use to purchase food items from a specified list: cabbage, green pepper, onion, tomato, egg, potato, carrot, mango, orange, and banana. The beneficiaries received social behaviour change communication (SBCC) messages about maternal infant and young child nutrition (MIYNC), anti-natal and postnatal care, and dietary diversity.

### 2.5. Data collection and analysis

Using the Unstructured Supplementary Service Data (USSD) technology, retailers set prices per unit (kg, bunch, and piece) for the food items they sold. Data on itemised food prices purchased by recipients was collected in real-time by the financial service provider (FSP) online system. Statistical analysis was completed using MS Excel and the Statistix 8.1 package. Descriptive statistics were used to observe the variation in mean values at a 5% level of significance.

## 3. Results

### 3.1. Food purchasing pattern of the beneficiaries

Overall, 51% of fresh food voucher beneficiaries purchased vegetables, 35% bought fruits, and only 15% purchased eggs. The three most popular food items were bananas, onions, and potatoes (Table 1). The high purchase of bananas may have been due to the taste preference of children, availability, and seasonality. Onion is part of every Ethiopian diet preparation as a condiment with low perishability. The high purchase of potatoes could be attributed to low perishability and low price.

**Table 1:** Relative purchase of food items by recipients.

Food items	*N* = 556	%
Banana	136	24
Onion	109	20
Potato	107	19
Eggs	81	15
Orange	57	10
Tomato	32	6
Carrot	17	3
Green pepper	9	2
Cabbage	7	1
Mango	1	0.1

### 3.2. Average food prices

The average price of fresh foods purchased by beneficiaries was 49 Ethiopian Birr (ETB; US$ 0.94; Table 2), albeit with different food items measured in different sizes. Fruits, on average, were the most expensive items, followed by egg and vegetables. Although orange, banana, and mango are not significantly different in terms of price, the beneficiaries tended to buy orange and banana rather than mango, perhaps due to the taste preference of children, seasonality, and the knowledge of mothers about the nutritional value of fruits, obtained from public health messages. Further evidence is needed regarding the behaviour of beneficiaries in terms of their purchases of different food items.

**Table 2:** Average food prices.^*^

Rank	Food item	Average price ETB (US$)
1	Orange	74 (2.6)
2	Banana	71 (2.1)
3	Mango	66 (2.1)
4	Eggs	65 (1.6)
5	Onion	63 (1.1)
6	Potato	48 (0.6)
7	Green Pepper	30 (0.3)
8	Cabbage	29 (0.2)
9	Carrot	23 (0.1)
10	Tomato	21 (0.02)
-	Average	49 (0.94)

^*^Portion sizes: eggs in dozens, cabbage in bunches, and all other items in kg.

### 3.3. Affordability of a nutritious diet

**Figure 1:**
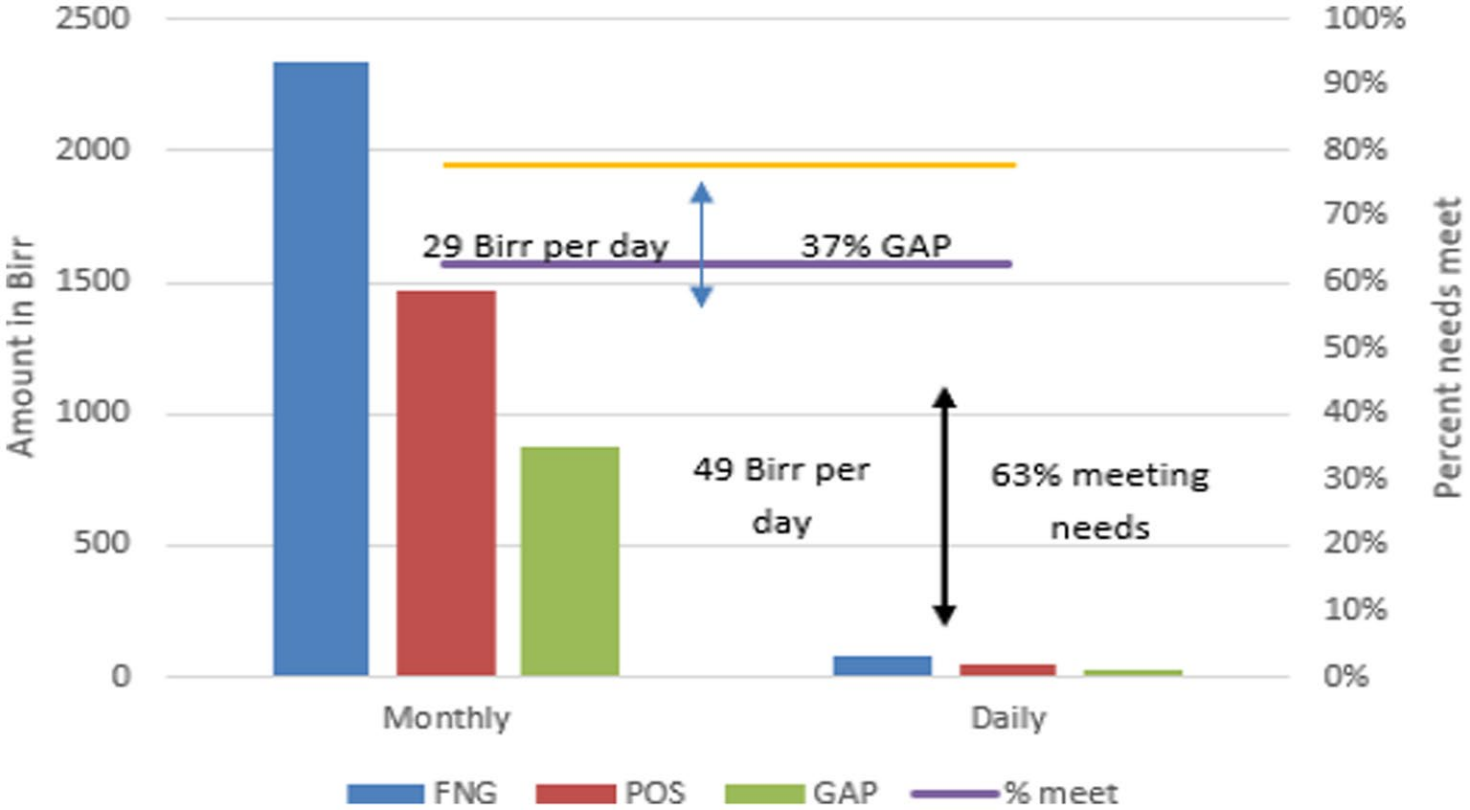
Average cost of nutritious diets (ETB): point of sale comparison of FNG and FFV.

Beneficiaries received 836 ETB (US$ 16.1) per month from the productive safety net programme (PSNP) and a similar amount from the WFP fresh food voucher programme (the exact amount depended on household size) to account for the non-affordability of a nutritious diet. Access to nutrient-dense food is limited in Ethiopia and households have limited affordability for a nutritious diet. The average household daily cost of a nutritious diet in Ethiopia is approximately 78 ETB (US$ 1.5). According to a previous study (6), the most vulnerable quintile of the population can only afford an average cost of 27 ETB (US$ 0.5) per day and 72% of vulnerable households remain unable to afford nutritious diets (Figure 1). The PSNP beneficiaries with in-kind support (digital voucher) or cash with a top-up from the WFP Fresh Food Voucher (FFV) programme experience a reduction in the non-affordability of a nutritious diet to 29 and 13%, respectively. The results from the pilot study indicate that the digital voucher for nutrient-dense food contributes to 63% of the daily (49 ETB, US$ 0.94) needs of targeted households.

### 3.4. Market food price variation

The collected data were evaluated for variations in fresh food prices that may directly affect the affordability of a diverse diet (Table 3). No significant differences were found in food item prices during the time frame except for cabbage, carrot, green pepper, onion, and tomato (*p* < 0.05). Further research is required to evaluate whether there is a negative impact on the affordability of a nutritious diet for targeted beneficiaries because of certain increased food prices. The increase in prices of certain food items might be due to the seasonality of production, which depends on rain-fed agriculture, but the exact reason needs to be investigated in future studies.

**Table 3:** Monthly food item prices (mean ± SD).

Sr.#	Food item/months	Sept	Oct	Nov	Dec	*p* value
1	Banana	34.60 ± 1.20	34.65 ± 0.87	36.60 ± 3.77	36.57 ± 3.73	3.646
2	Cabbage	14.13 ± 5.85	13.52 ± 4.98	16.14 ± 3.38	11.52 ± 2.14	**0.001**
3	Carrot	10.07 ± 7.17	10.90 ± 8.35	13.26 ± 11.68	0.0	**0.014**
4	Egg	5.11 ± 0.16	5.15 ± 0.17	5.01 ± 0.01	5.35 ± 0.18	3.124
5	Green pepper	14.58 ± 6.48	11.82 ± 2.57	18.33 ± 11.78	0.0	**0.005**
6	Mango	44.50 ± 3.54	41.25 ± 1.77	41.50 ± 2.12	0.0	8.110
7	Onion	22.62 ± 1.68	27.67 ± 1.35	35.08 ± 3.60	35.43 ± 4.10	**0.001**
8	Orange	48.81 ± 5.39	34.70 ± 16.55	33.64 ± 15.04	21.75 ± 29.34	0.090
9	Potato	22.94 ± 0.08	22.68 ± 0.95	23.44 ± 2.14	22.71 ± 1.12	4.195
10	Tomato	24.83 ± 2.92	30.15 ± 4.61	30.17 ± 4.59	0.0	**0.001**

Bold value means significance difference.

## 4. Discussion

The findings reveal that fruits and vegetables were consumed in larger quantity, 34 and 51% respectively, indicating the high intake of carbohydrates, vitamins, and minerals. Bananas (24%) in the fruits category and onions (20%) and potatoes (19%) in the vegetables category were highly purchased food items by the Fresh Food Voucher (FFV) beneficiaries. The high purchase of potatoes and bananas may be related to the lower price and frequent use in Ethiopian dish sauce for potato and the preference of children for bananas. The high need for and purchase of onions was expected as they are frequently used in condiments in traditional Ethiopian cooking. However, egg consumption, which was the only protein-rich food source in the digital voucher, was only 15%.

Food purchasing is considered a key mediator between the food environment and eating behaviour. Food purchasing patterns are increasingly measured in epidemiologic and intervention studies. However, the extent to which food purchases reflect an individual’s dietary intake has not been rigorously tested (8).

The average price of fresh foods purchased by recipients was 49 ETB (US$ 0.94; Table 2), albeit with different food items measured in different sizes. Fruits, on average, were the most expensive items, followed by eggs and vegetables. The impact of high food prices on nutrition begins with households and individuals. As household purchasing power goes down, dietary quality and total energy intake are reduced, compromising both child growth and maternal nutrition (9).

The food purchases reflect proxy dietary intake, i.e., that people eat what they buy, and their consumption therefore reflects the quality and nutrient density of the purchased food items. However, the relative contributions of foods consumed away from home, food waste, and consumption by other household members may affect the degree to which episodes of food purchasing reflect actual dietary intake (10). Further assessment is needed to determine the effect of purchasing patterns on the dietary diversity of the targeted households.

## 5. Conclusion

Results of the analysis show that some food items are preferred over others. Vegetables were purchased more than fruits by beneficiaries. The driving force behind the greater purchase of certain food items over others is subject to further research. The WFP Fresh Food Voucher (FFV) programme contributed 63% of the daily (49 ETB, US$ 0.94) affordability needs of the targeted households for a nutritious diet. The remaining 37% (1,503 ETB, US$ 28.9) gap per month would need to be met through additional income or support. Using the point of sale (POS) system enabled by Unstructured Supplementary Service Data (USSD) technology as a pilot was an effective means of collecting real-time data on itemised food items and prices and allowed the timely monitoring of the project, which facilitated its accountability.

## 6. Recommendations

- The Unstructured Supplementary Service Data (USSD) technology should be used in full to gather real-time data for programme course correction.
- To obtain a full picture of the Fresh Food Voucher (FFV) intervention, further research should consider the food utilisation aspect, which may include dietary diversity, seasonality, and certain food purchasing behaviours of the beneficiaries.
- Scaling up the point of sale system in the Fresh Food Voucher (FFV) programme would enable more retailers to record data on itemised food prices and allow for wider analysis of the purchasing patterns of beneficiaries.
- The data collected through the POS system will enable researchers to determine the beneficiaries’ purchasing patterns of different fresh foods and will support the timely social behaviour change communication (SBCC) strategy to maximise the impact of the Fresh Food Voucher (FFV) programme on the dietary diversity in children under 2 years of age and pregnant and lactating women.

## 7. Limitations

The limitations in the implementation of the POS system were a lack of technological knowledge, skills, and interest of beneficiaries and vendors, redemption time out of the POS system, and mobile phone network coverage in the area.

## Data availability statement

The original contributions presented in the study are included in the article/supplementary material, further inquiries can be directed to the corresponding authors.

## Author contributions

All authors listed have made a substantial, direct, and intellectual contribution to the work and approved it for publication.

## Footnotes

^2^Fill the Nutrient Gap (FNG) is a collaborative analytical process (a secondary literature review in combination with linear optimisation) to understand local drivers that affect the availability, cost, and affordability of nutritious diets. It aims to strengthen analysis, build consensus, and improve decision-making to make healthy and nutritious diets widely available.
